# *QuickStats*: Percentage* of Currently Employed Adults^†^ with No Health Insurance,^§^ by Type of Work Arrangement^¶^ — National Health Interview Survey, 2010 and 2015**

**DOI:** 10.15585/mmwr.mm6632a9

**Published:** 2017-08-18

**Figure:**
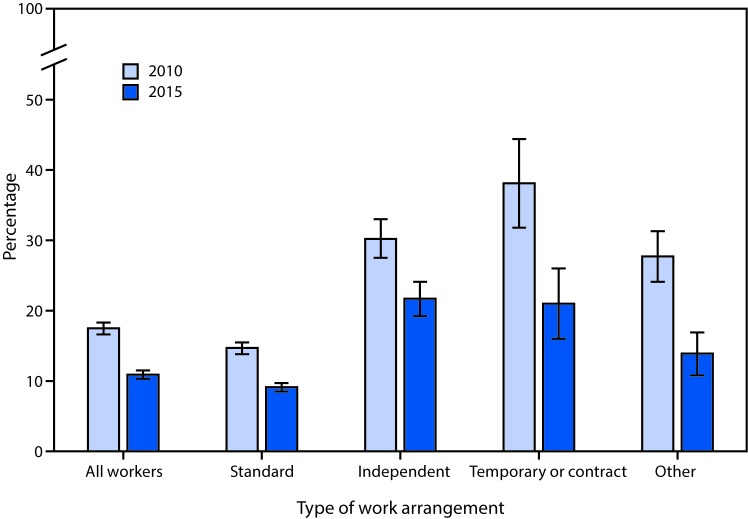
The percentage of all workers with no health insurance decreased from 17.5% in 2010 to 10.9% in 2015. The percentage also declined in each type of work arrangement. In 2015, independent workers (21.7%) or temporary/contract workers (21.0%) were more likely to lack health insurance than workers with a standard work arrangement (9.1%).

**Source:** National Health Interview Survey. https://www.cdc.gov/nchs/nhis/index.htm.

